# Haemorrhoidal artery ligation versus rubber band ligation for the management of symptomatic second-degree and third-degree haemorrhoids (HubBLe): a multicentre, open-label, randomised controlled trial

**DOI:** 10.1016/S0140-6736(16)30584-0

**Published:** 2016-07-23

**Authors:** Steven R Brown, James P Tiernan, Angus J M Watson, Katie Biggs, Neil Shephard, Allan J Wailoo, Mike Bradburn, Abualbishr Alshreef, Daniel Hind, The HubBLe Study team

**Affiliations:** a Sheffield Teaching Hospitals, Sheffield, UK; b St James's University Hospital, Leeds, UK; c Raigmore Hospital, Inverness, UK; d Clinical Trials Research Unit, School of Health and Related Research, University of Sheffield, Sheffield, UK; e Health Economics and Decision Science, School of Health and Related Research, University of Sheffield, Sheffield, UK

## Abstract

**Background:**

Optimum surgical intervention for low-grade haemorrhoids is unknown. Haemorrhoidal artery ligation (HAL) has been proposed as an efficacious, safe therapy while rubber band ligation (RBL) is a commonly used outpatient treatment. We compared recurrence after HAL versus RBL in patients with grade II–III haemorrhoids.

**Methods:**

This multicentre, open-label, parallel group, randomised controlled trial included patients from 17 acute UK NHS trusts. We screened patients aged 18 years or older presenting with grade II–III haemorrhoids. We excluded patients who had previously received any haemorrhoid surgery, more than one injection treatment for haemorrhoids, or more than one RBL procedure within 3 years before recruitment. Eligible patients were randomly assigned (in a 1:1 ratio) to either RBL or HAL with Doppler. Randomisation was computer-generated and stratified by centre with blocks of random sizes. Allocation concealment was achieved using a web-based system. The study was open-label with no masking of participants, clinicians, or research staff. The primary outcome was recurrence at 1 year, derived from the patient's self-reported assessment in combination with resource use from their general practitioner and hospital records. Recurrence was analysed in patients who had undergone one of the interventions and been followed up for at least 1 year. This study is registered with the ISRCTN registry, ISRCTN41394716.

**Findings:**

From Sept 9, 2012, to May 6, 2014, of 969 patients screened, 185 were randomly assigned to the HAL group and 187 to the RBL group. Of these participants, 337 had primary outcome data (176 in the RBL group and 161 in the HAL group). At 1 year post-procedure, 87 (49%) of 176 patients in the RBL group and 48 (30%) of 161 patients in the HAL group had haemorrhoid recurrence (adjusted odds ratio [aOR] 2·23, 95% CI 1·42–3·51; p=0·0005). The main reason for this difference was the number of extra procedures required to achieve improvement (57 [32%] participants in the RBL group and 23 [14%] participants in the HAL group had a subsequent procedure for haemorrhoids). The mean pain 1 day after procedure was 3·4 (SD 2·8) in the RBL group and 4·6 (2·8) in the HAL group (difference −1·2, 95% CI −1·8 to −0·5; p=0·0002); at day 7 the scores were 1·6 (2·3) in the RBL group and 3·1 (2·4) in the HAL group (difference −1·5, −2·0 to −1·0; p<0·0001). Pain scores did not differ between groups at 21 days and 6 weeks. 15 individuals reported serious adverse events requiring hospital admission. One patient in the RBL group had a pre-existing rectal tumour. Of the remaining 14 serious adverse events, 12 (7%) were among participants treated with HAL and two (1%) were in those treated with RBL. Six patients had pain (one treated with RBL, five treated with HAL), three had bleeding not requiring transfusion (one treated with RBL, two treated with HAL), two in the HAL group had urinary retention, two in the HAL group had vasovagal upset, and one in the HAL group had possible sepsis (treated with antibiotics).

**Interpretation:**

Although recurrence after HAL was lower than a single RBL, HAL was more painful than RBL. The difference in recurrence was due to the need for repeat bandings in the RBL group. Patients (and health commissioners) might prefer such a course of RBL to the more invasive HAL.

**Funding:**

NIHR Health Technology Assessment programme.

## Introduction

Haemorrhoids result from enlargement of the haemorrhoidal plexus and pathological changes in the anal cushions, a normal component of the anal canal. They are common, affecting about a third of the population.^1^ Approximately 23 000 haemorrhoidal operations were done in England in 2004–05.^2^ Repeated visits to hospital for therapy represent an important disruption to personal and working lives.

Treatment depends on the degree of symptoms and prolapse, ranging from dietary advice, outpatient rubber band ligation (RBL), to operation requiring anaesthesia. Although RBL is cheap and serious complications rare, recurrence is common, particularly where prolapse is substantial.^3^ Patients often require further banding.^3^ Although variations exist (eg, ligasure haemorrhoidectomy), surgery is usually traditional haemorrhoidectomy or a stapled haemorrhoidopexy, both requiring anaesthesia. Traditional haemorrhoidectomy is associated with considerable postoperative discomfort, sometimes necessitating admission to hospital and delayed return to normal activity, but recurrence is low. Stapled haemorrhoidopexy has a slightly higher recurrence rate but patients return to normal activity more quickly than with traditional haemorrhoidectomy.^4^

**Research in context**

**Evidence before this study**

Haemorrhoidal artery ligation (HAL) is a relatively new procedure that has become increasingly established as a treatment for haemorrhoids. A NICE overview in 2010 and four systematic reviews published between 2009 and 2015 highlight the lack of good quality data as evidence for the advantages of the HAL technique. The reviews included five randomised trials, two comparative cohort trials, and 21 cohort studies. From these reviews, the pooled recurrence rate for HAL ranged from 11% to 17·5%. A commonly used technique for treatment of early grade haemorrhoids is rubber band ligation (RBL). This technique is simple and easy to carry out, requiring no anaesthetic and with rapid recovery. It is the obvious comparator for treatment of early grade haemorrhoids. To date, there have been no randomised trials that have compared HAL with RBL.

**Added value of this study**

We did a multicentre, parallel-group randomised controlled trial of 370 patients comparing HAL with RBL. The recurrence rate for HAL was significantly lower than for RBL (30% *vs* 49%, p=0·001) at 12 months. Further treatment was required in 31% of the RBL group and 15% of the HAL group (adjusted odds ratio [aOR] for further procedure 2·86, 95% CI 1·65–4·93; p=0·0002). 18% of the RBL group required a second banding session within the year. Excluding these patients as recurrence if they reported improvement or cure at 1 year resulted in a larger reduction of our recurrence rate for RBL and no statistical difference between the groups (HAL 30% *vs* RBL 37·5%, aOR 1·35, 0·85–2·15; p=0·20). Quality of life, symptom severity score, continence score, and complications occurred at a similar frequency. Pain was greater and lasted longer after an HAL procedure. The health-care cost analysis was striking. In the base case results, HAL was around £1000 more expensive and is highly unlikely to be cost-effective at the £20 000–30 000 threshold.

**Implications of all the available evidence**

The results of this study suggest both procedures have a higher recurrence rate than previously reported. Although the recurrence rate at 1 year is lower for HAL compared with a single RBL, many clinicians would consider RBL as a course of treatment. If those with repeat RBL within the year are excluded as recurrence if they reported cure or improvement at 1 year, the recurrence rate was similar. Other outcomes were also similar or worse (in terms of pain) after HAL. HAL is more expensive and not cost-effective.

An alternative treatment is haemorrhoidal artery ligation (HAL). Although requiring anaesthesia, evidence suggests a recovery similar to RBL, but an effectiveness that approaches the more intensive surgical options. As a consequence, the HAL procedure has gained popularity, with more than 5000 procedures carried out in the UK per year (manufacturer communication).

Estimates of HAL efficacy come from several randomised trials, four systematic reviews,^5^, ^6^, ^7^, ^8^ and one overview by the National Institute for Health and Care Excellence (NICE).^9^ All publications highlight the lack of good quality data as evidence for the advantages of the technique. To our knowledge, there are no existing randomised trials comparing HAL with RBL.

We aimed to establish the clinical and cost-effectiveness of HAL compared with RBL in the treatment of symptomatic second-degree and third-degree haemorrhoids. The primary objective was to compare patient-reported symptom recurrence 12 months after intervention.

## Methods

The protocol was published in 2012;^10^ protocol amendments subsequent to trial commencement are provided in the appendix.

### Study design and participants

This multicentre, parallel-group randomised controlled trial took place in 17 acute UK NHS hospitals. Delegated study staff at these 17 hospitals identified and consented potential participants. Eligible participants were aged 18 years or older with symptomatic second-degree or third-degree haemorrhoids.^11^ We excluded patients who had previously received any haemorrhoid surgery, more than one injection treatment for haemorrhoids, or more than one RBL procedure within 3 years before recruitment. We also excluded patients with perianal sepsis, inflammatory bowel disease, colorectal malignancy, pre-existing sphincter injury, and immunodeficiency, hypercoagulability disorders, and patients who were unable to have general or spinal anaesthetic. Sheffield CTRU coordinated follow-up and data collection in collaboration with these centres. The study was approved by the NRES Committee South Yorkshire (REC reference 12/YH/0236). All participants provided written informed consent.

### Randomisation and masking

Participants were individually randomly assigned (in a 1:1 ratio) to receive either HAL or RBL. Randomisation was computer-generated and stratified by centre using permuted blocks of random sizes two, four, and six. Allocation concealment was achieved using a centralised web-based randomisation system in which the participant identifier was entered before the allocation was revealed. The study was open-label with no blinding of participants, clinicians, or research staff.

### Procedures

The pre-randomisation and baseline questionnaires included EQ-5D, pain visual analogue scale (VAS), Vaizey faecal incontinence score, and the Haemorrhoid Symptom Severity (HSS) score. Baseline data collected before the procedure (usually on the day of surgery) included ethnic origin, smoking history, height, weight, comorbidities, grade of haemorrhoid, and previous treatments for haemorrhoids.

RBL is performed with a device that applies a rubber band to each haemorrhoid via a proctoscope. This band constricts the blood supply causing it to become ischaemic before being sloughed off 1–2 weeks later. The resultant fibrosis reduces any prolapse that might be present. The procedure is a basic surgical skill that all senior staff within the NHS are familiar with and competent in performing. Bands were applied at the discretion of the surgeon but with a view to resolution of all disease.

HAL is performed with a proctoscope modified to incorporate a Doppler transducer. There are two types of equipment in common use, the HALO device (AMI HAL Doppler system, CJ Medical, Truro, UK) and the THD device (THD Lab, Correggio, Italy). Both devices enable accurate detection and targeted suture ligation of the haemorrhoidal arteries. When combined with a so-called pexy suture, both bleeding and haemorrhoidal prolapse are addressed. All surgeons participating in the trial ensured the need for a pexy suture due to prolapse was routinely assessed and recorded. The procedure is simple, uses existing surgical skills, and has a short learning curve, with the manufacturers recommending at least five mentored cases before independently practising. All surgeons involved in the study had completed this training and had carried out an additional five independent procedures before recruitment.

Day 1, 7, and 21 questionnaires were given to the participants following their procedure and data were either collected over the telephone or the questionnaire was returned by post or handed in at the 6 week visit. These questionnaires included EQ-5D and a pain VAS.

Questionnaires at 6 weeks were collected at the clinic visit (or over the telephone if there was no visit); these included EQ-5D, pain VAS, Vaizey score, HSS, and questions regarding further treatment.

The clinical assessment form was completed at the 6 week visit by the consultant or from patient notes. If a proctoscopy was completed, this information was recorded here, along with a recurrence question (same as the primary outcome question) data on any complications, further treatment and planned treatment.

To identify the proportion of patients with recurrent haemorrhoids at 12 months after intervention, since no validated patient-reported symptom score exists, we asked participants a question, 12 months post-intervention:^12^ “At the moment, do you feel your symptoms from your haemorrhoids are: (1) cured or improved compared with before treatment; or (2) unchanged or worse compared with before treatment?”

Patients were considered to have recurrent haemorrhoids when any of the following were recorded: “unchanged or worse compared with before starting treatment” at 12 months, patient reported; “any subsequent procedure” (RBL, HAL, haemorrhoidectomy, haemorrhoidopexy, haemorrhoidal injection or other relevant procedure) over the 12 months (general practitioner [GP] and/or hospital records); or presence of any symptoms or events that strongly indicate recurrence (among patients not meeting the two previous criteria), as adjudicated by two blinded trial investigators (JPT, SRB; appendix).

Other data collected from questionnaires at 12 months were: EQ-5D, pain VAS, Vaizey score, HSS, and questions regarding further treatment.

Postoperative assessment was included in the protocol, but was not carried out universally. If patients said they were better, many surgeons did not re-examine. This practice is in line with current majority clinical practice.

Complementary, adjunctive treatments (eg, dietary counselling, stool hygiene and habits, use of fibre, use of local therapies such as vasoconstrictors) were not specifically included in the trial and were prescribed at surgeon's discretion along the lines of the pragmatic study design.

### Outcomes

The primary outcome was the proportion of patients with recurrent haemorrhoids at 12 months after procedure, derived from the patient's self-reported assessment in combination with resource use from their GP and hospital records.

Secondary endpoints assessed at 6 weeks and 12 months were: symptom severity (assessed with an HSS adapted from Nyström and colleagues),^13^ incontinence inventories (assessed using the validated Vaizey faecal incontinence score),^10^ pain (assessed using a 10 cm VAS), surgical complications, need for further treatment, persistent symptoms at 6 weeks, and health-state utility based on the EQ-5D.^10^

### Statistical analysis

Assuming the proportion of patients who experience recurrence after RBL is 30% and after HAL is 15%, the sample size required for 80% power and 5% significance was 121 individuals per group. To account for any between-surgeon variation and loss to follow-up, this number was increased to 175 per group, on the basis of a 10% attrition and a conservative assumption that there would be 14 surgeons in the trial and an intraclass correlation (ICC) of 2·5% in keeping with typical ICCs.^14^ However, we considered it likely that each site would have at least two surgeons, in which case the power to detect this difference was 85%; 90% power if there was no between-surgeon variation. Because the surgical procedure was well developed and standardised, ICC was expected to be virtually zero and we expected the proposed sample size to have closer to 90% power.

We did the primary analyses in individuals who had undergone one of the interventions and been followed up for at least 1 year (defined as the study population in text). We did additional analyses in the per-protocol (PP) population, restricted to those individuals who complied with the protocol. Deviations from the protocol that were not considered in relation to PP analysis were related to the consent process, missed windows for the assessments, eligibility (two leading to amending the exclusion criteria and two ineligible participants were withdrawn), and one participant was given general anaesthetic for the RBL procedure.

We did the analysis of recurrence using a random intercept logistic regression model in which covariates were treatment allocation, sex, age at surgery, and history of previous intervention as fixed effects; the surgeon was included as a random effect. Further sensitivity analyses assessed whether other baseline characteristics (symptom score, EQ-5D-5L, body-mass index) altered the strength or appeared to modify the treatment effect. We compared the severity of haemorrhoidal symptoms between groups using a generalised least squares regression model, with the same covariates as the primary outcome. We did some sensitivity analyses that adjusted for severity at randomisation (where available), at baseline, and the average of the two. The difference in symptom severity was compared separately for the 6-week and 12-month timepoints. We analysed EQ-5D-5L, incontinence, and pain in the same manner as symptoms. We compared descriptively the secondary outcome of complications elicited during the complications review interview or from the patient notes at 6 weeks and 1 year after intervention. A planned analysis of the time to recurrence was dropped because of the difficulty of eliciting the time of patient-reported recurrence.

All confidence intervals were two-sided 95% intervals comparing HAL to RBL and all statistical hypotheses were two-sided tests. We did a cost-utility analysis in terms of incremental cost per quality-adjusted life-years (QALYs) gained. We calculated the costs (including repeat procedures) following the standard three stage approach: identification of resource use, measurement, and valuation using the National NHS reference costs. We did a secondary cost-effectiveness analysis, which estimated incremental cost per recurrence avoided. All costs were estimated from the NHS and personal social perspective as per NICE recommendations.^15^ Analyses were undertaken using the R and Stata programs.

This project will be published in full in the *Public Health Research* journal series. This trial is registered (ISRCTN41394716).

### Role of the funding source

The funder of the study had no role in study design, data collection, data analysis, data interpretation, or writing of the report. The corresponding author had full access to all the data in the study and had final responsibility for the decision to submit for publication.

## Results

**Figure 1:**
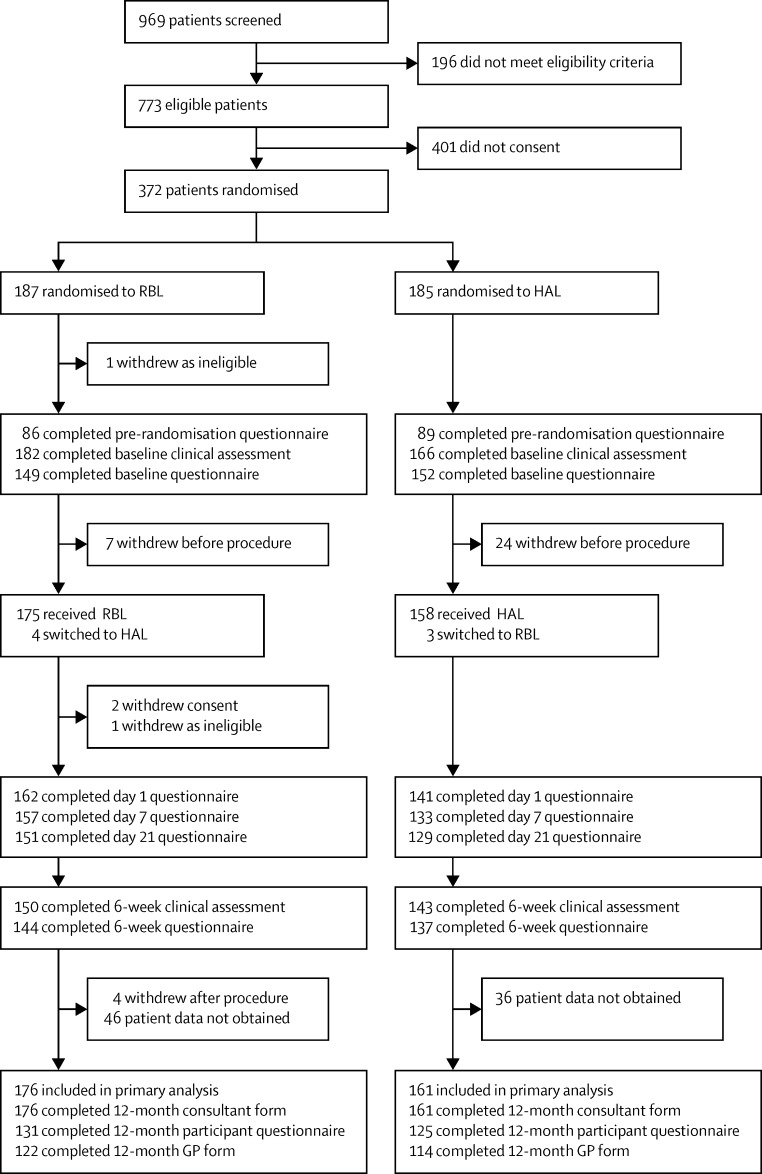
Trial profile RBL=rubber band ligation. HAL=haemorrhoidal artery ligation.

Between Sept 9, 2012, and May 6, 2014, 372 participants (of the 969 screened) were randomly assigned to receive RBL or HAL; 187 participants were allocated to receive RBL, and 185 were allocated to receive HAL (figure 1). Two of these participants (both randomly assigned to RBL) were removed from the trial completely due to ineligibility: one before the procedure, and the second after the procedure. Of the 370 randomised participants who were followed up, 340 received treatment (figure 1); their baseline characteristics are shown in table 1.

340 participants received treatment (figure 1). Primary outcome data were available for 337 participants (161 in the HAL group and 176 in the RBL group). At 12 months, 256 fully completed patient questionnaires, 236 GP forms, and 337 consultant forms were returned. Follow-up was completed at sites on Aug 28, 2015. The median time from surgery to follow-up was 367 days (365–385) for the RBL group and 367 days (365–374) for the HAL group. There were 183 participants for whom all three of the 12 months forms were fully completed and returned (98 in the RBL group and 88 in the HAL group). The analysis population included all 176 participants in the RBL group and 161 participants in the HAL group for whom recurrence data were available, from either the patient, clinician, or GP. Four participants received HAL despite being randomly assigned to RBL, whereas three participants assigned to HAL received RBL. Since the findings in the primary analysis population and the PP population were similar, the reporting is restricted to the primary analysis population, with the exception of adverse events and complications which are by treatment received.

The number of participants with a recurrence at 12 months was 87 (49%) in the RBL group compared with 48 (30%) in the HAL group (adjusted odds ratio [aOR] 2·23, 95% CI 1·42–3·51, ICC=0·000; p=0·0005). The breakdown of recurrences, overall and by criteria is presented in table 2. The proportion of participants who reported recurrence was similar between groups, with 29% of respondents in both groups stating they believed symptoms from their haemorrhoids were unchanged or worse (aOR for self-reported recurrence 1·06, 0·60–1·85; p=0·85). The increased recurrence associated with RBL was mainly attributable to the high rate of additional procedures undertaken following initial intervention (32%), compared with 14% in the HAL group by 1 year follow-up (aOR for further procedure 2·86, 1·65–4·93; p=0·0002). A further three (2%) participants in the RBL group were considered to have symptoms consistent with recurrent haemorrhoids following review of medical contacts and procedures over the 12 month follow-up, which were undertaken blind to treatment group: in two cases the participants were recorded as possibly requiring further treatment at their 6-week visit but were subsequently lost to follow-up; a third had been admitted to hospital twice for excessive bleeding but had not undergone treatment.

At 6 weeks, data were available for 293 patients (figure). 43 (29%) of 150 participants in the RBL group reported their haemorrhoids as unchanged or worse, compared with 12 (8%) of 143 participants in the HAL group; additionally, one participant in each group had subsequently undergone RBL. Thus the overall number of patients with persistent symptoms was 44 (29%) versus 13 (9%); adjusted odds ratio 4·35 (95% CI 2·19–8·65; p<0·0001).

At 6 weeks, HSS scores were higher in the RBL group, indicating short-term symptoms were less pronounced following HAL (appendix). The mean scores were 4·0 (SD 3·5) in the RBL group and 3·0 (3·1) in the HAL group, with an adjusted difference in means of 1·0 (95% CI 0·3 to 1·8; p=0·010). No difference was apparent at 12 months, with the mean being 3·6 (3·2) for RBL and 3·6 (3·3) for HAL (adjusted difference 0·0, 95% CI −0·8 to 0·8; p=0·98).

**Figure 2:**
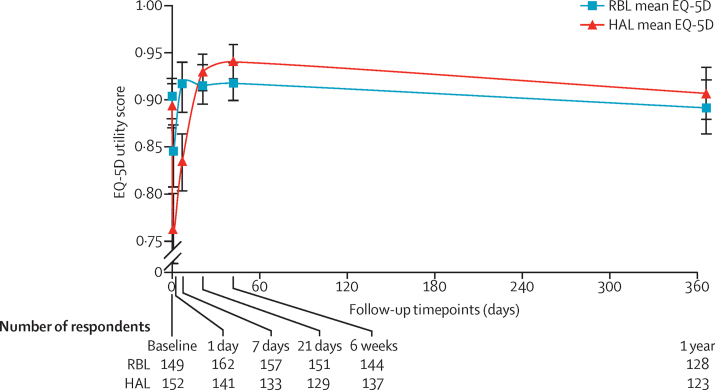
Mean EQ-5D scores with 95% CIs over 12 months follow-up Some participants did not complete all measures in the forms, five participants (three in the RBL group, two in the HAL group) did not complete the EQ-5D score measure. RBL=rubber band ligation. HAL=haemorrhoidal artery ligation.

Before intervention, the mean health utility (EQ-5D-5L) was around 0·9 in both groups but declined at days 1 and 7 in the HAL group (figure 2). For RBL the mean at day 1 was 0·84 (SD 0·19) and at day 7 it was 0·92 (0·15); in other words, health state was reduced for the first day but had reverted back at 1 week. By contrast, the mean health state for HAL had not returned to baseline values by day 7, with the mean being 0·76 (0·22) at day 1 and 0·83 (0·18) at day 7. The adjusted difference in means were 0·08 (95% CI 0·04–0·13; p<0·001) at day 1 and 0·08 (0·05–0·12; p=0·001) at day 7. The mean health utility was nearly similar with no statistical differences between the two groups (and above baseline values) at all timepoints from day 21 onwards.

The Vaizey faecal incontinence score was similar between groups (appendix). An improvement of around one unit was noted in both groups at 6 weeks, with a difference between groups of −0·1 (95% CI −1·3 to 1·0; p=0·86). The improvement was maintained at 1 year, with a difference of 0·5 (95% CI −0·7 to −1·8; p=0·38).

Patients rated their current pain due to haemorrhoids at baseline and at four timepoints over the subsequent 6 weeks using a 10-point VAS. HAL was associated with more short-term pain than was RBL. The mean pain 1 day after procedure was 3·4 (SD 2·8) in the RBL group and 4·6 (2·8) in the HAL group (difference −1·2, 95% CI −1·8 to −0·5; p=0·0002); at day 7 the mean scores were 1·6 (2·3) in the RBL group and 3·1 (2·4) in the HAL group (difference −1·5, −2·0 to −1·0; p<0·0001). The mean pain was similar between groups at 21 days (1·3 [2·0] in the RBL group *vs* 1·4 [1·9] in the HAL group; difference −0·1, −0·6 to 0·3; p=0·44) and 6 weeks (1·2 [2·1] in the RBL group *vs* 1·0 [1·8] in the HAL group; difference 0·2, −0·2 to 0·7; p=0·32).

15 individuals reported serious adverse events requiring hospital admission (table 3). One patient experienced several episodes of bleeding after RBL; further investigations revealed a rectal tumour. This serious adverse event was classified as pre-existing and was not included. Of the remaining 14 serious adverse events, 12 (7%) were among participants treated with HAL (one of whom had been switched from the RBL group) and two (1%) were in those treated with RBL. Six patients had pain requiring prolonged hospital stay (five treated with HAL, one treated with RBL), three had bleeding (not requiring transfusion, two treated with HAL, one treated with RBL), two had urinary retention, two had vasovagal upset, and one had possible sepsis (treated with antibiotics); all 14 events were prespecified as expected in the study protocol.

The main findings of within trial cost-utility analysis suggest that the HAL procedure appeared not to be cost-effective compared with RBL at a cost-effectiveness threshold of £20 000–30 000 per QALY. In the base-case results, the difference in mean total costs was £1027 higher for HAL than for RBL (table 4). QALYs were higher for HAL than for RBL; however, the difference was very small (0·010), resulting in an incremental cost-effectiveness ratio (ICER) of £104 427 per additional QALY. At £20 000 per QALY threshold, HAL has zero probability of being cost-effective; at £30 000 threshold, it has 0·05 probability of being cost-effective.

The mean total cost per patient for HAL was £1750 (95% CI 1333–2167) compared with £723 (551–896) for RBL (table 3).

Among the 80 participants who required a further procedure, the majority (15 of 23 in the HAL group and 45 of 57 in the RBL group) underwent a single procedure and in most cases this was RBL, although some variation was noted across centres: the cost of an additional RBL procedure in this trial was £523·16. As RBL is a brief outpatient procedure with (relatively) minimal inconvenience to the patient, it could be argued that a repeat RBL is not itself indicative of a recurrence. Consequently, we did an additional post-hoc analysis to investigate the extent to which recurrence differed between an outpatient course of RBL treatment and HAL (ie, excluding second bandings in the RBL group). Of the 31 patients in the RBL group who underwent repeat RBL, 21 were reclassified as non-recurrences since they reported being cured or improved at 1 year. This analysis changed the number of recurrences to 66 (37·5%) in the RBL group, and 48 (30%) in the HAL group (adjusted odds ratio 1·35, 95% CI 0·85–2·15; p=0·20).

A further (post-hoc) analysis looked at the proportion of participants whose symptom score was either zero or one, since this number corresponds to the definition of cure used by Nyström.^13^ The proportions suggest that although there were more “cured” patients in the HAL group at 6 weeks (31% RBL *vs* 38% HAL) and 1 year (27% RBL *vs* 31% HAL), there was no statistical difference between the groups (adjusted OR 0·73, 0·44–1·22; p=0·23 at 6 weeks and OR 0·79, 0·46 to 1·38; p=0·42 at 1 year).

The trial design meant that there was a difference between interventions in terms of dates of randomisation and surgery (RBL was often carried out immediately whereas HAL patients went onto a waiting list; see table 1). Because of this, baseline data were recorded both at randomisation and at the time of surgery. The extent of agreement was generally similar regardless of the severity.

## Discussion

Recurrence 12 months after HAL was significantly lower than after RBL. Haemorrhoidal disease is a benign condition with treatment primarily aimed at addressing symptoms. In the absence of a validated symptom scoring system, we felt the most important determinant of treatment success was patient-reported outcome of improvement and the need to avoid additional procedures. Where patients had undergone further intervention for haemorrhoids, they were considered to have recurred. Based on this premise, HAL appears superior. This apparent superiority should be put in practical context. 18% of the participants in the RBL group underwent repeat banding. This is common practice and patients might find this re-banding a more palatable option than having an operation if it has the same potential for improvement. Indeed some clinicians deem RBL as a course of treatment. Including these patients as a success (if they reported improvement at 12 months) resulted in a reduction in recurrence and no statistical difference between the groups.

The choice of patient-defined recurrence as our primary outcome was supported by a robust patient and public involvement exercise carried out during trial design. Clinical experience indicates that the physical appearance of the anal cushions post-treatment correlates poorly with patients' symptoms, meaning anorectal visualisation is not a reliable surrogate of success.^16^ We therefore asked participants a question, 12 months after intervention, based on a simple, dichotomised definition of recurrence as described by Shanmugam and colleagues.^12^ This definition is not universal—indeed no standardised definition of recurrence exists. This is the most obvious explanation for our recurrence rates following HAL, higher than those reported in systematic reviews (11–17·5%).^5^, ^6^ Some previous studies have relied on clinical examination and patient-reported symptoms,^17^, ^18^, ^19^, ^20^, ^21^ others solely on symptomatology.^22^, ^23^ When considering symptoms only, some investigators consider a patient recurrence-free if they have no perianal symptoms at all; others simply an improvement. An example of variation dependent on this definition is provided by one study reporting a recurrence rate of 60%, 30 months after a HAL procedure, yet 86% of patients simultaneously described a significant improvement in symptoms.^22^ Other factors that influence recurrence include duration of follow-up. We chose to assess recurrence at 12 months because published data suggest most recurrences occur in less than 1 year.^19^ However, our data indicate that persistent symptoms were lower for both groups at 6 weeks (HAL 9% *vs* RBL 29%) with major deterioration over the year and it is not clear if or when this deterioration plateaus.

The mean symptom severity score improved after both interventions by 2 to 3 points on a 15 point scale at 6 weeks. Accounting for other variables in the model (age, gender, previous treatment, and baseline score), those undergoing RBL had a higher HSS score at this timepoint but no difference was apparent at 1 year. The improvement after both interventions was not as great as that seen by Nyström and colleagues who observed a difference of about 6 points after intervention.^12^ However, in that study the preoperative score was higher than in ours, reflecting a higher grade of haemorrhoids pre-treatment.

Applying the Nyström definition of cure as 0–1 point on this scoring system,^13^ there was no difference between the two interventions at any timepoint. Indeed, it is striking how few patients reported such a score despite considering themselves to be cured or improved according to our primary outcome measure. This finding suggests that many patients are not concerned with a certain level of persistent symptoms related to haemorrhoids.

Intervention for haemorrhoids is essentially aimed at improving quality of life, which therefore becomes an important indicator of success. Our results suggest that the majority of patients in both groups had an improvement in quality of life above baseline after intervention from day 21 onwards. Before this date there was a difference in favour of RBL, probably related to the fact that the HAL procedure was more painful and pain lasted longer. Although no long-term difference was seen between the two groups, both interventions did result in a small improvement in quality-of-life score. Therefore, both interventions appear worthwhile from this perspective. The alternative management option of reassurance following exclusion of a sinister cause for the symptoms and giving general lifestyle advice might not be appropriate.^24^

Resting anal sphincter tone maintains continence and the haemorrhoidal tissue contributes to the formation of a hermetic seal. It follows that disruption of this tissue due to prolapse potentially leads to anal leakage. Our results are consistent with this: at baseline the majority of participants from both groups had no or very mild incontinence. Correction of the prolapse resulted in a small reduction in the mean continence score after both interventions with no difference between interventions.

Most patients reported pain following both procedures. For RBL this was usually of low intensity, and resolved rapidly to below baseline with approximately half of patients requiring analgesics for a few days. For HAL, the pain was significantly greater up to 1 week but had resolved in almost all patients by 3 weeks. Daily analgesia was required by most for the first week but tailed off, such that at 3 weeks three-quarters of patients had stopped taking medication. There are few randomised trials where VAS scores have been used as we have used them; summarising these findings suggests most patients have moderate pain in the first few days after an HAL, but that this pain recedes to minimal or no pain by 1–3 weeks,^22^, ^23^, ^25^ consistent with our results.

The incidence of adverse events was low for both treatment groups. Two patients from the RBL group required hospitalisation. This is relevant as the procedure is usually carried out in the outpatient department, often with minimal consent. There is a vogue for some hospitals to arrange for the RBL procedure to be carried out in a day-case theatre environment, allowing a formal consent process. Day-case admission, with a short period of observation, may have avoided the need for hospital admission in only one patient in our trial who had immediate severe pain post-procedure.

The health-care cost analysis is striking. In the base case analysis, HAL is around £1000 more expensive than RBL. Since there is little difference in overall health-related quality of life between the two procedures, the ICER per additional QALY is very high and significantly exceeds a cost-effectiveness threshold of £20 000–30 000 per QALY. Even if a difference in recurrence is assumed (ie, single RBL procedure *vs* HAL) the cost-effectiveness ratio in terms of cost per recurrence avoided is approximately £5000. Essentially, HAL is highly unlikely to be cost-effective at the £20 000–30 000 per QALY threshold.

Data regarding health-care costs from other studies is sparse. Cost analysis has been carried out in one trial comparing stapled haemorrhoidopexy with RBL,^26^ with the cost of stapled haemorrhoidopexy being substantially higher and unlikely to be considered cost-effective at 1 year. However, the authors assumed that the cost due to increased recurrence with RBL would mean that this difference would fall over time. This assumption might be true for HAL, although the relative low cost of RBL makes it unlikely. It is probably more cost-effective for patients to return for a further RBL if recurrence occurs at any time.

We noted more patients who withdrew in the HAL group than in the RBL group. The main reason for the difference in withdrawal relates to patients withdrawing consent. 15 patients from the HAL group withdrew consent compared with two from the RBL group. This difference inevitably relates to the waiting time for intervention. RBL was often carried out immediately after randomisation whereas for HAL patients were put on a waiting list. In some instances the waiting list was very long (up to 270 days). There were otherwise no differences in the baseline characteristics for those that withdrew.

The pragmatic, multicentre design, using a mix of NHS district general and teaching hospitals across the UK, ensures that the results are generalisable to all patients seeking treatment for grade II–III haemorrhoids. However, there are potential limitations. First, the length of follow-up might not be adequate; extended follow-up might demonstrate further deterioration in symptoms, altering health-care costs. Second, we used the Goligher grading system for haemorrhoids, which has been criticised for its definition of grade IV haemorrhoids;^16^ there is less ambiguity with regard to lower grades but misclassification is common.^27^ Third, although HAL appears simple and easy to learn, there might be a prolonged learning curve. Some studies describe a poorer outcome in initial patients.^28^, ^29^ Our trial required that surgeons had to have carried out at least five mentored cases and an additional five procedures before recruitment (based on manufacturer recommendations), but no data exist to define when competence is reached. The final limitation is the lack of a validated scoring system for haemorrhoids. At the time of design of this trial there were no such systems available. Subsequent to our study starting, a scoring system has been developed.^30^

In conclusion, HAL is more effective than single RBL. If, however, RBL is considered a course of treatment involving repeat banding, the procedures are equally effective. Similarly, symptom severity score, complications, quality of life, and continence score are no different between HAL and RBL, and patients had more pain in the early postoperative period after HAL. HAL is significantly more expensive and unlikely to be cost-effective. Because of this, patients (and commissioners) might prefer a course of RBL for the treatment of haemorrhoids.

**This online publication has been corrected. The corrected version first appeared at thelancet.com on July 21, 2016**

## Figures and Tables

**Table 1:** Baseline demographic data by randomised group

	**RBL (n=176)**	**HAL (n=161)**
**Sex**		
Male	99 (56%)	85 (53%)
Female	77 (44%)	76 (47%)
**Age, years**		
Mean (SD)	49·0 (12·9)	48·5 (13·5)
Median (IQR)	50·5 (38·5–58·0)	49·0 (38·0–60·0)
Range	21·9–79·3	20·2–74·6
**Body-mass index, kg/m^2^**		
Mean (SD)	28·0 (557)	28·2 (7·1)
Median (IQR)	27·0 (24·4–31·7)	26·8 (24·1–30·0)
Range	17·4–44·9	18·8–67·4
**Grade of haemorrhoids**		
II (%)	115 (65%)	92 (57%)
III (%)	60 (34%)	68 (42%)
Missing	1 (0·6%)	1 (0·6%)
**Previous treatment**		
No (%)	124 (70%)	124 (70%)
Yes (%)	52 (30%)	36 (22%)
Missing	0	1 (0·6%)
**Procedure information**		
Number of patients receiving procedure	172 (98%)	158 (98%)
Median time from randomisation to procedure, days (IQR)	0 (0–19)	62 (39–91)
Number of patients receiving treatment on the same day as randomisation	114 (63%)	0

Data are n (%) or median (IQR). RBL=rubber band ligation. HAL=haemorrhoidal artery ligation.

**Table 2:** Recurrence at 12 months after procedure

			**RBL (n=176)**	**HAL (n=161)**
Recurrence			87 (49%)	48 (30%)
Criteria for recurrence*				
	Self-reported recurrence		37/130 (29%†)	34/124 (29%†)
	Subsequent procedure for haemorrhoids		57 (32%)	23 (14%)
		RBL	31 (18%)	14 (9%)
		HAL	23 (13%)	7 (4%)
		Excisional haemorrhoidectomy	4 (2%)	7 (4%)
		Stapled haemorrhoidopexy	2 (1%)	1 (1%)
	Residual untreated symptoms from blinded review		3 (2%)	0

RBL=rubber band ligation. HAL=haemorrhoidal artery ligation.

* More than one criterion may apply.

† Denominator is the number of patients returning the questionnaire (or form).

**Table 3:** Serious adverse events

		**RBL group (n=178)**	**HAL group (n=162)**
Any serious adverse events		2 (1%)	12 (7%)
	Excessive bleeding	0	3 (2%)
	Urinary retention	0	2 (1%)
	Sepsis	0	1 (<1%)
	Pain requiring hospital admission	1	5*
	Vasovagal upset	1	1

RBL=rubber band ligation. HAL=haemorrhoidal artery ligation.

* Includes one patient randomly assigned to RBL, but who received HAL.

**Table 4:** The mean difference in total costs and QALYs for HAL versus RBL

	**RBL**	**HAL**	**Mean difference (SE)**	**95% CI**	**p value**
Costs (£)	723	1750	1027 (124)	782–1272	<0·0001
QALYs	0·919	0·924	0·01 (0·01)	−0·02–0·04	0·4890

RBL=rubber band ligation. HAL=haemorrhoidal artery ligation. QALY=quality-adjusted life-years.
