# Department of Error

**DOI:** 10.1016/S0140-6736(16)31108-4

**Published:** 2016-07-23

*Brown SR, Tiernan JP, Watson AJM, et al. Haemorrhoidal artery ligation versus rubber band ligation for the management of symptomatic second-degree and third-degree haemorrhoids (HubBLe): a multicentre, open-label, randomised controlled trial.* Lancet *2016; **388:** 356–64*—In this Article, the results for mean EQ-5D utility score should have been: “For RBL the mean at day 1 was 0·84 (SD 0·19) and at day 7 it was 0·92 (0·15)…with the mean being 0·76 (0·22) at day 1 and 0·83 (0·18) at day 7. The adjusted difference in means were 0·08 (95% CI 0·04–0·13; p<0·001) at day 1 and 0·08 (0·05–0·12; p<0·001) at day 7. The mean health utility was nearly similar with no statistical differences between the two groups (and above baseline values) at all timepoints from day 21 onwards.” In the Discussion section, the following sentence has been clarified: “Even if a difference in recurrence is assumed, (ie, single RBL procedure vs HAL) the cost-effectiveness in terms of cost per recurrence avoided is approximately £5000”. This correction has been made to the online version as of July 21, 2016, and the printed version is correct.

