# Supplementary material for: Haemorrhoidal artery ligation versus rubber band ligation for the management of symptomatic second-degree and third-degree haemorrhoids (HubBLe): a multicentre, open-label, randomised controlled trial
Source: Lancet. 2016 Jul 23;388(10042):356–64. doi: 10.1016/S0140-6736(16)30584-0 (PMC4956910; doi:10.1016/S0140-6736(16)30584-0)
Supplement: Supplementary appendix [file mmc1.pdf]

THE LANCET

Supplementary appendix

This appendix formed part of the original submission and has been peer reviewed. We post it as supplied by the authors.

Supplement to: Brown SR, Tiernan JP, Watson AJM, et al, and The HubBLe Study team. Haemorrhoidal artery ligation versus rubber band ligation for the management of symptomatic second-degree and third-degree haemorrhoids (HubBLe): a multicentre, open-label, randomised controlled trial. *Lancet* 2016; published online May 25. http://dx.doi.org/10.1016/S0140-6736(16)30584-0.

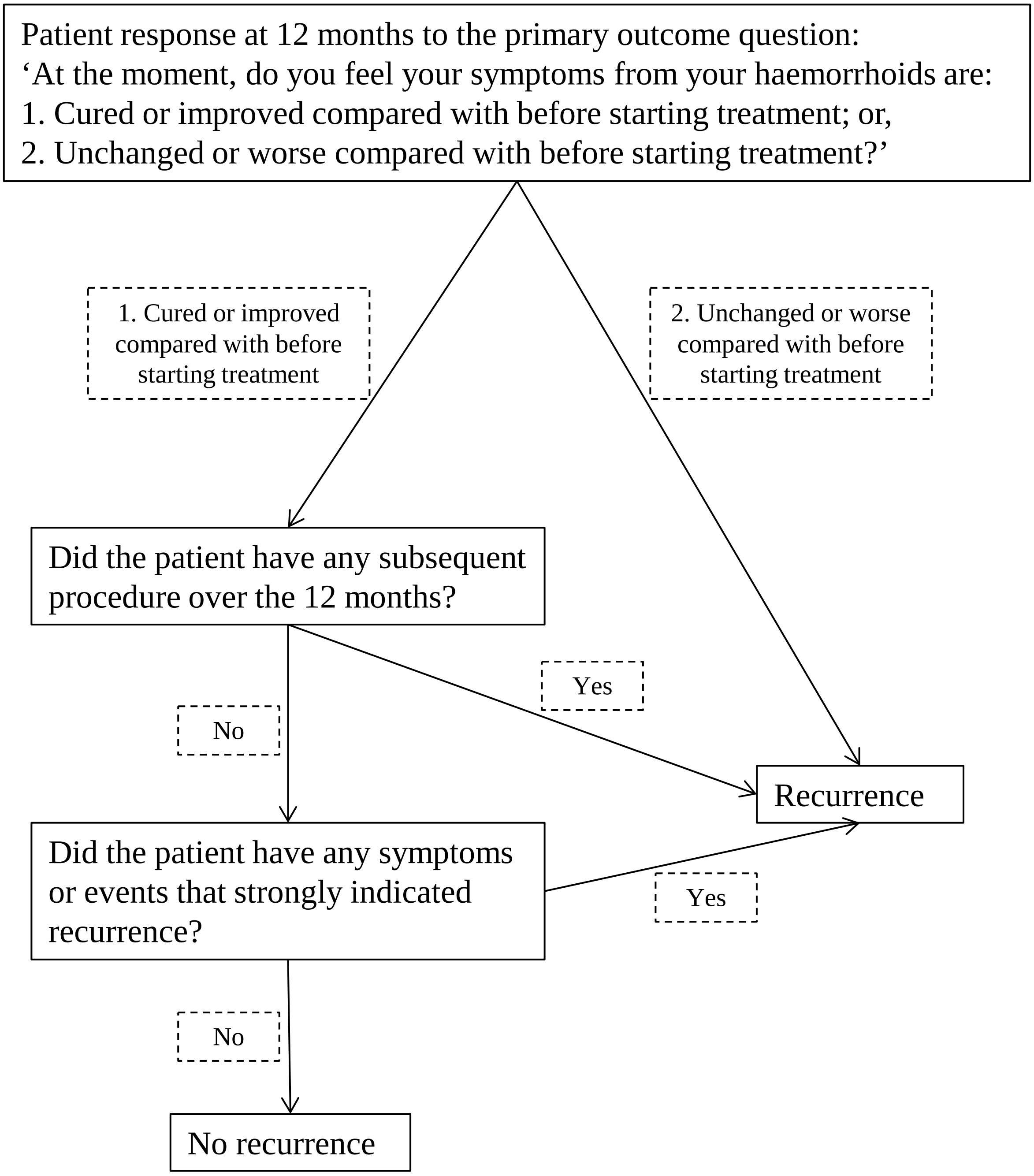
Patient response at 12 months to the primary outcome question:
'At the moment, do you feel your symptoms from your haemorrhoids are:
1. Cured or improved compared with before starting treatment; or,
2. Unchanged or worse compared with before starting treatment?'
1. Cured or improved compared with before starting treatment
2. Unchanged or worse compared with before starting treatment
Did the patient have any subsequent procedure over the 12 months?
Yes
No
Recurrence
Did the patient have any symptoms or events that strongly indicated recurrence?
Yes
No
No recurrence

Web-only information Table 1a – Changes to protocol during trial commencement

Changes to Protocol	Date	Approved by
Protocol version 2.0: Adverse Events were clarified in the protocol and participant information sheet	30/07/2012	NRES Committee Yorkshire & The Humber - South Yorkshire
Protocol version 3.0: Clarified Serious Adverse Event reporting to state that only related Adverse Events and Serious Adverse Events will be reported	30/08/2012	NRES Committee Yorkshire & The Humber - South Yorkshire
Protocol version 4.0: A change was made to the eligibility criteria to exclude patients with hyper-coagulability disorders (in addition to those on Warfarin or Clopidogrel)	24/10/2012	NRES Committee Yorkshire & The Humber - South Yorkshire
Protocol version 5.0: The baseline data collection was changed to the day of surgery, rather than at randomisation.	07/02/2013	NRES Committee Yorkshire & The Humber - South Yorkshire
Protocol version 6.0: The inclusion criteria ‘Either presenting for the first time or after one failure of RBL’ was replace by three exclusion criteria for clarification: ‘Patients that have had previous surgery for haemorrhoids (at any time); Patients that have had more than one injection treatment for haemorrhoids in the past 3 years; and Patients that have had more than one RBL procedure in the past 3 years’	25/03/2013	NRES Committee Yorkshire & The Humber - South Yorkshire
Protocol version 7.0: The sentence 'patients will have at least 24 hours to decide whether to take part' was added to the protocol, to provide clarification.	30/04/2013	NRES Committee Yorkshire & The Humber - South Yorkshire
Protocol version 8.0: A pre-randomisation questionnaire was added to the data collection schedule for participants	05/07/2013	NRES Committee Yorkshire & The Humber - South Yorkshire

Web-only information Table 2a - Haemorrhoid symptom scores

	RBL	HAL	Difference (95% CI)
Randomisation			
n	83	80	
Mean (SD)	6.5 (3.2)	6.4 (3.4)	
Median (IQR)	6.0 (4.0 to 8.5)	6.0 (4.0 to 9.0)	
Baseline			
N	146	150	
Mean (SD)	6.5 (3.3)	6.4 (3.0)	
Median (IQR)	6.0 (4.0 to 8.0)	6.0 (4.0 to 8.5)	
6 weeks			
n	142	137	
Mean (SD)	4.0 (3.5)	3.0 (3.1)	1.0 (0.3 to 1.8); p=0.01
Median (IQR)	3.0 (1.0 to 6.0)	2.0 (1.0 to 5.0)	
12 months			
n	131	123	
Mean (SD)	3.6 (3.2)	3.6 (3.3)	0.0 (-0.8, 0.8); p=0.98
Median (IQR)	3.0 (1.0 to 5.0)	3.0 (1.0 to 5.2)	

Web only information – Vaizey faecal incontinence scores

	RBL	HAL	Difference (95% CI)’ p-value
Randomisation			
N	84	88	
Mean (SD)	6.0 (5.3)	4.9 (4.6)	
Median (IQR)	4.0 (1.0 to 10.0)	4.0 (2.0 to 7.5)	
Baseline			
N	142	147	
Mean (SD)	5.9 (5.2)	5.3 (4.8)	
Median (IQR)	4.0 (1.0 to 9.0)	4.0 (2.0 to 8.0)	
6 weeks			
N	137	132	
Mean (SD)	4.0 (4.5)	4.1 (4.9)	-0.1 (-1.3 to 1.0); p = 0.86
Median (IQR)	3.0 (0.0 to 6.0)	3.0 (0.0 to 6.0)	
1 year			
N	118	107	
Mean (SD)	4.0 (4.7)	4.5 (4.5)	-0.5 (-1.8 to 0.7); p = 0.38
Median (IQR)	2.0 (0.0 to 6.0)	3.0 (1.0 to 7.0)	
